# Phytosterol accumulation results in ventricular arrhythmia, impaired cardiac function and death in mice

**DOI:** 10.1038/s41598-021-96936-x

**Published:** 2021-08-31

**Authors:** Hongfei Ge, Gongxin Liu, Tracy M. Yamawaki, Caroline Tao, Shawn T. Alexander, Kimberly Ly, Preston Fordstrom, Artem A. Shkumatov, Chi-Ming Li, Sridharan Rajamani, Mingyue Zhou, Brandon Ason

**Affiliations:** 1 Cardiometabolic Disorders Therapeutic Area, Amgen Research, Amgen, Inc, 1120 Veterans Blvd, South San Francisco, CA 94080 USA; 2 Genomic Analysis Unit, Amgen Research, South San Francisco, CA USA; 3 Translational Safety and Bioanalytical Sciences, Amgen Research, South San Francisco, CA USA

**Keywords:** Cardiovascular biology, Target identification

## Abstract

Heart failure (HF) and cardiac arrhythmias share overlapping pathological mechanisms that act cooperatively to accelerate disease pathogenesis. Cardiac fibrosis is associated with both pathological conditions. Our previous work identified a link between phytosterol accumulation and cardiac injury in a mouse model of phytosterolemia, a rare disorder characterized by elevated circulating phytosterols and increased cardiovascular disease risk. Here, we uncover a previously unknown pathological link between phytosterols and cardiac arrhythmias in the same animal model. Phytosterolemia resulted in inflammatory pathway induction, premature ventricular contractions (PVC) and ventricular tachycardia (VT). Blockade of phytosterol absorption either by therapeutic inhibition or by genetic inactivation of NPC1L1 prevented the induction of inflammation and arrhythmogenesis. Inhibition of phytosterol absorption reduced inflammation and cardiac fibrosis, improved cardiac function, reduced the incidence of arrhythmias and increased survival in a mouse model of phytosterolemia. Collectively, this work identified a pathological mechanism whereby elevated phytosterols result in inflammation and cardiac fibrosis leading to impaired cardiac function, arrhythmias and sudden death. These comorbidities provide insight into the underlying pathophysiological mechanism for phytosterolemia-associated risk of sudden cardiac death.

## Introduction

Phytosterolemia is a rare genetic disorder caused by mutations to ABCG5/8, a heterodimeric ABC sterol transporter responsible for blocking intestinal absorption of dietary cholesterol and phytosterols as well as the removal of cholesterol and phytosterols from circulation by promoting their transport into bile for secretion into the intestinal lumen^1^. It is characterized by elevated circulating phytosterols such as β-sitosterol, campesterol and stigmasterol and a remarkably high risk of cardiovascular disease^2–5^. Phytosterols are solely acquired from dietary sources such as vegetable oil, soybeans, nuts and seeds. Despite being present at comparable quantities as cholesterol in a typical human diet, phytosterols are largely prevented from entering circulation by ABCG5/8 expressed in the intestine as they are effectively excreted through bile via ABCG5/8^6–8^. Its function is essential as rare ABCG5/8 loss of function variants cause phytosterolemia and increase the risk of developing coronary artery disease, experiencing a myocardial infarction or sudden cardiac death^9–16^.

Niemann-Pick C1-Like 1 (NPC1L1) is the opposing sterol transporter^17–19^. While ABCG5/8 is responsible for dietary sterol excretion, NPC1L1 is responsible for the absorption of dietary cholesterol and phytosterols. NPC1L1 and ABCG5/8 together control dietary sterol input to maintain cholesterol and phytosterol homeostasis.

Phytosterolemia is considered a recessive disorder, requiring either homozygous or compound heterozygous inactivation of the ABCG5/8 transporter for disease manifestation. However, recent studies indicate that heterozygous carriers can exhibit a milder form of the disease^12,20^. To date, only a small number of patients have been diagnosed with phytosterolemia. However, 1 out of every 200,000 individuals are predicted to carry loss of function mutations in either AGCG5 or ABCG8 suggesting that phytosterolemia may be widely overlooked^21,22^. This may be attributed to the fact that phytosterolemia patients can be misdiagnosed with hypercholesterolemia, as standard analytical methods used to quantify cholesterol are unable to distinguish cholesterol from structurally related phytosterols^23^.

Phytosterolemia related cardiovascular outcomes are frequently attributed to coronary artery disease, a disease characterized by elevated circulating cholesterol (hypercholesterolemia) resulting in atherosclerosis even though clinical manifestations of phytosterolemia are notably distinct from hypercholesterolemia. To our knowledge, there are no known reports that exclude hypercholesterolemia or provide evidence of either cardiac damage or arrhythmias that contributed to cardiovascular outcomes for patients with phytosterolemia. However, it is worth noting that the ABCG5/8 rare coding variant, His250Tyr, was recently found to be associated with aortic valve stenosis, heart failure and sudden cardiac death^9^.

Sudden cardiac death is frequently attributed to arrhythmia and is commonly found to be associated with myocardial fibrosis^24–26^. Cardiac fibrosis is strongly considered as both a cause and a consequence of arrhythmias and HF^10,26,27^. Cardiac fibrosis can manifest in patients with rare inflammatory diseases such as sarcoidosis resulting in congestive HF, cardiac arrhythmias, or both^28^. The severity of cardiac fibrosis correlates with the incidence and progression of both HF and arrhythmias^27,29^. Patients with HF are predisposed to developing cardiac arrhythmias including complex ventricular arrhythmias, ventricular tachycardia and atrial fibrillation^30,31^. Likewise, cardiac arrhythmias predispose individuals to HF and contribute to worsening HF prognosis. This relationship between HF and cardiac arrhythmias is driven by numerous shared co-morbidities and overlapping pathologies.

We recently demonstrated that phytosterolemia can cause cardiac fibrosis, impair cardiac function and was associated with premature death in mice^23^. Here, we show that genetic inactivation or pharmacological inhibition of NPC1L1, which blocks intestinal absorption of phytosterols, compensated for the loss of ABCG5/8 function by preventing phytosterol induced inflammation and cardiac fibrosis, improved cardiac function, ablated ventricular arrhythmias and increased survival. Bulk RNA sequencing of cardiac tissue revealed that phytosterol accumulation was associated with an induction of inflammatory response genes as well as genes associated with arrhythmias. These disease-associated changes in gene expression were absent in ezetimibe treated mice, concurrent genetic inactivation of NPC1L1, or both. Taken together, our findings identify a previously unknown link between phytosterolemia, arrhythmia and sudden cardiac death in mice.

## Results

### Genetic inactivation of NPC1L1 corrects phytosterolemia induced inflammation, cardiac fibrosis, cardiac dysfunction and the incidence of death caused by ABCG5/8 deficiency

**Figure 1:**
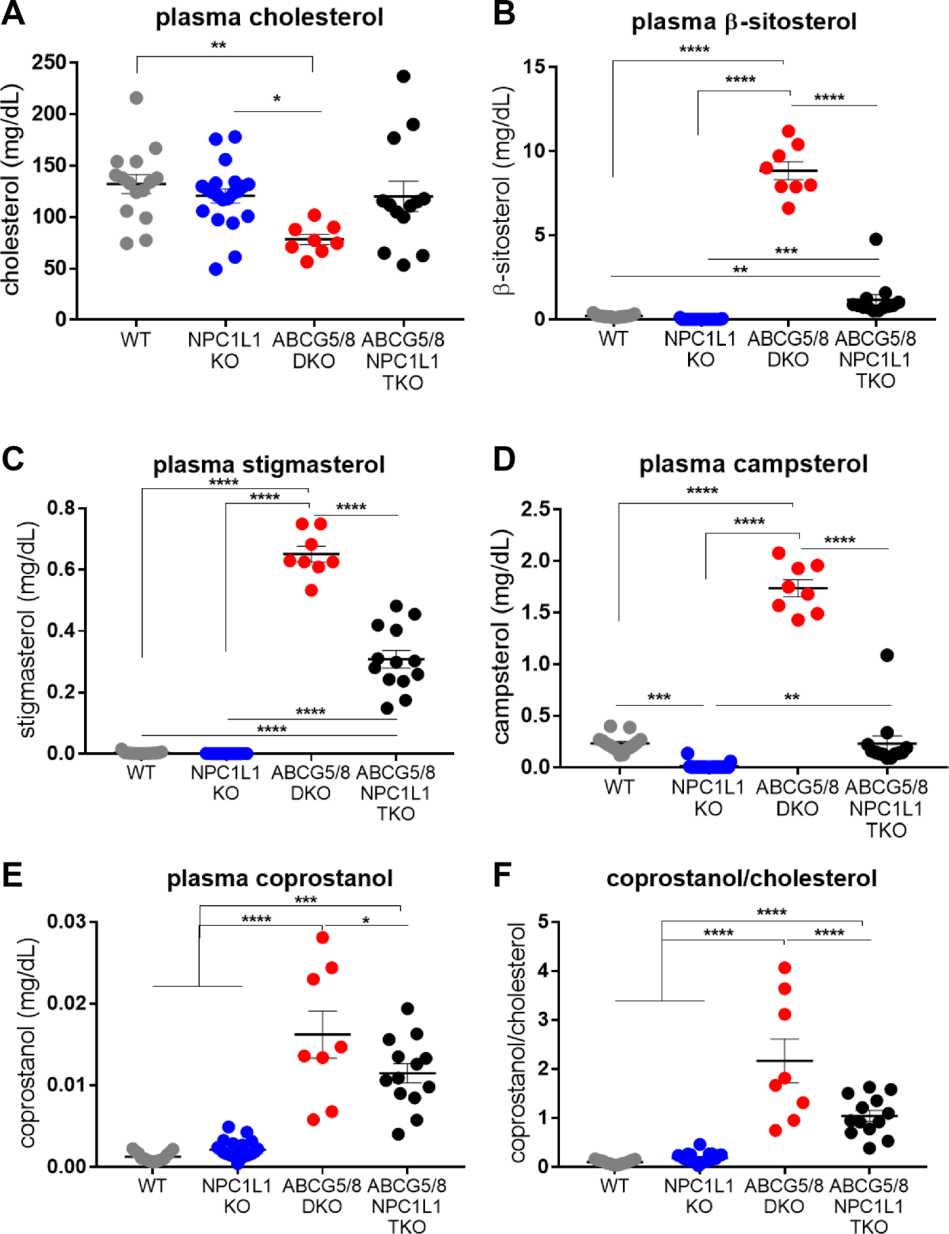
The NPC1L1, ABCG5/8 TKO exhibits a reduction in circulating phytosterols relative to ABCG5/8 DKO littermates. ABCG5/8 DKO exhibit reduced plasma cholesterol (**A**) and increased phytosterols (β-sitosterol (**B**), stigmasterol (**C**), campsterol (**D**), coprostanol (**E**)) as well as the coprostanol/cholesterol ratio (**F**). Circulating phytosterols accumulated in TKO mice in comparison to WT littermates but at much lower levels relative to ABCG5/8 DKO littermates. DKO—ABCG5/8 double knockout, TKO—ABCG5/8, NPC1L1 triple knockout. Individual data points (circles) are shown together with the sample mean (bar) ± SEM. Significance measured by one-way ANOVA—Tukey post-test (*P < 0.05; **P < 0.01; ***P < 0.001; ****P < 0.0001).

Our previous work demonstrated that phytosterolemia induced by feeding ABCG5/8 double knockout (DKO) mice a phytosterol enriched diet led to cardiac fibrosis and death^23^. It also revealed that three weeks of ezetimibe treatment reduced both plasma phytosterols and associated cardiac fibrosis^23^. Here, we utilized NPC1L1 knockout (KO) mice to determine if reducing phytosterol absorption by genetic inactivation could relieve phytosterolemia associated pathologies, including mortality^32^. Mice that were heterozygous KOs for both ABCG5/8 and NPC1L1 were crossed to each other (Het/Het x Het/Het) to produce, among others, the following cohorts used for our study: (1) wild type, (2) NPC1L1 homozygous KOs, (3) ABCG5/8 homozygous DKOs, and (4) NPC1L1, ABCG5/8 homozygous triple knockout (TKO) littermates. The animals produced from this cross were between 99 and 100% congenic to the C57Bl/6J background line. Twenty-week old littermates were placed on the phytosterol enriched diet (chow supplemented with 0.2% stigmasterol, 0.2% sitosterol) for 7 weeks. Sterol concentrations were determined by LC–MS/MS to differentiate cholesterol from the individual phytosterols. ABCG5/8 DKO mice fed a phytosterol enriched diet exhibited a significant increase in plasma phytosterols relative to wild type or NPC1L1 KO littermate controls (Fig. 1A–F). NPC1L1, ABCG5/8 TKO mice exhibited a significant reduction in phytosterol levels relative to ABCG5/8 DKO littermates. Interestingly, plasma β-sitosterol, stigmasterol, coprostanol and the coprostanol/cholesterol ratio in the NPC1L1, ABCG5/8 TKOs were significantly higher relative to the NPC1L1 KO and wild type littermates indicating that NPC1L1 inactivation is unable to fully counteract ABCG5/8 deficiency.

**Figure 2:**
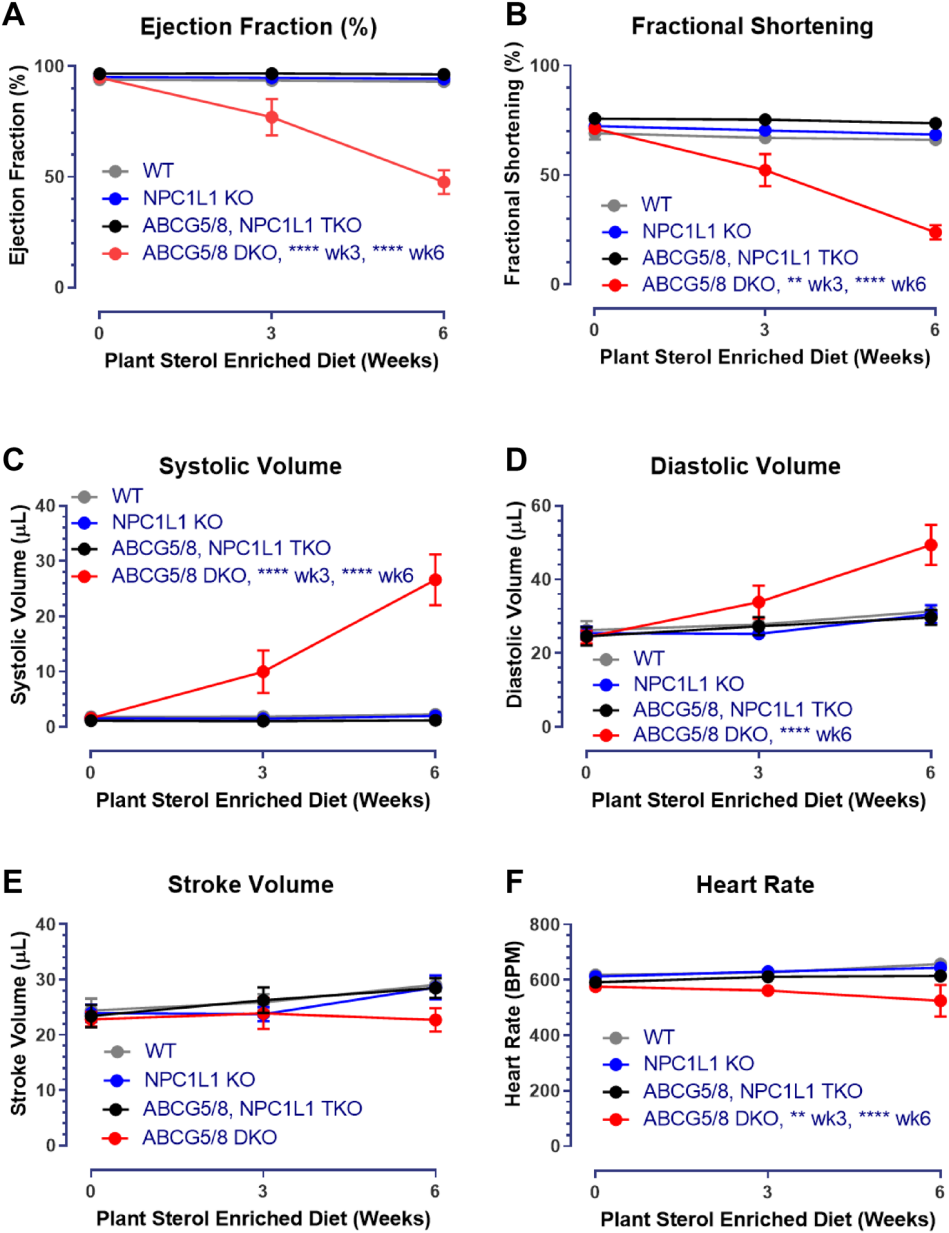
The NPC1L1, ABCG5/8 TKO exhibits improved cardiac function relative to ABCG5/8 DKO littermates. ABCG5/8 DKO mice exhibit decreased ejection fraction (**A**) and fractional shortening (**B**) and an increase in both systolic (**C**) and diastolic volume (**D**) with negligible changes to stroke volume (**E**) and a modest decrease in heart rate (**F**), which is indicative of impaired cardiac function and left ventricular dilation. These impairments in cardiac function are not observed for the TKO, NPC1L1 KO or WT littermate controls. DKO—ABCG5/8 double knockout, TKO—ABCG5/8, NPC1L1 triple knockout. Data represented as group means (circles) ± SEM. WT: N = 15/ group; NPC1L1 KO: N = 20–21/group; TKO: N = 11–13/group; DKO: N = 6–12/group. Significance measured using a mixed-effects model (REML)—Tukey’s multiple comparisons test (*P < 0.05; **P < 0.01; ***P < 0.001; ****P < 0.0001 relative to WT littermates). Data analyses performed blinded.

We next assessed cardiac function using echocardiography. Left ventricular function was compared between conscious littermates at baseline, 3- and 6-weeks following placement on the phytosterol enriched diet. For all groups, echocardiographic end points such as left ventricular ejection fraction (EF) were relatively high at baseline due to the animals being conscious and possibly stressed during data acquisition. Cardiac function declined for ABCG5/8 DKO mice at week 3 and worsened further 6 weeks relative to the other groups following the switch to the phytosterol enriched diet as evident by a significant decrease in EF, fractional shortening (FS) and an increase in end systolic volume (ESV) and end diastolic volume (EDV) (Fig. 2A–D). This was associated with a modest decrease in stroke volume (SV) and heart rate (HR) for the ABCG5/8 DKOs compared to littermates (Fig. 2E,F). There was no difference in the heart to brain weight ratios between groups after 7 weeks indicating that LV dilation was not associated with a change in heart mass (Supplemental Fig. 1). The phytosterol induced decline in cardiac function was prevented through the simultaneous inactivation of NPC1L1 and ABCG5/8 as TKO mice exhibited measures of cardiac function that were comparable to both wild type and NPC1L1 KO littermate controls.

**Figure 3:**
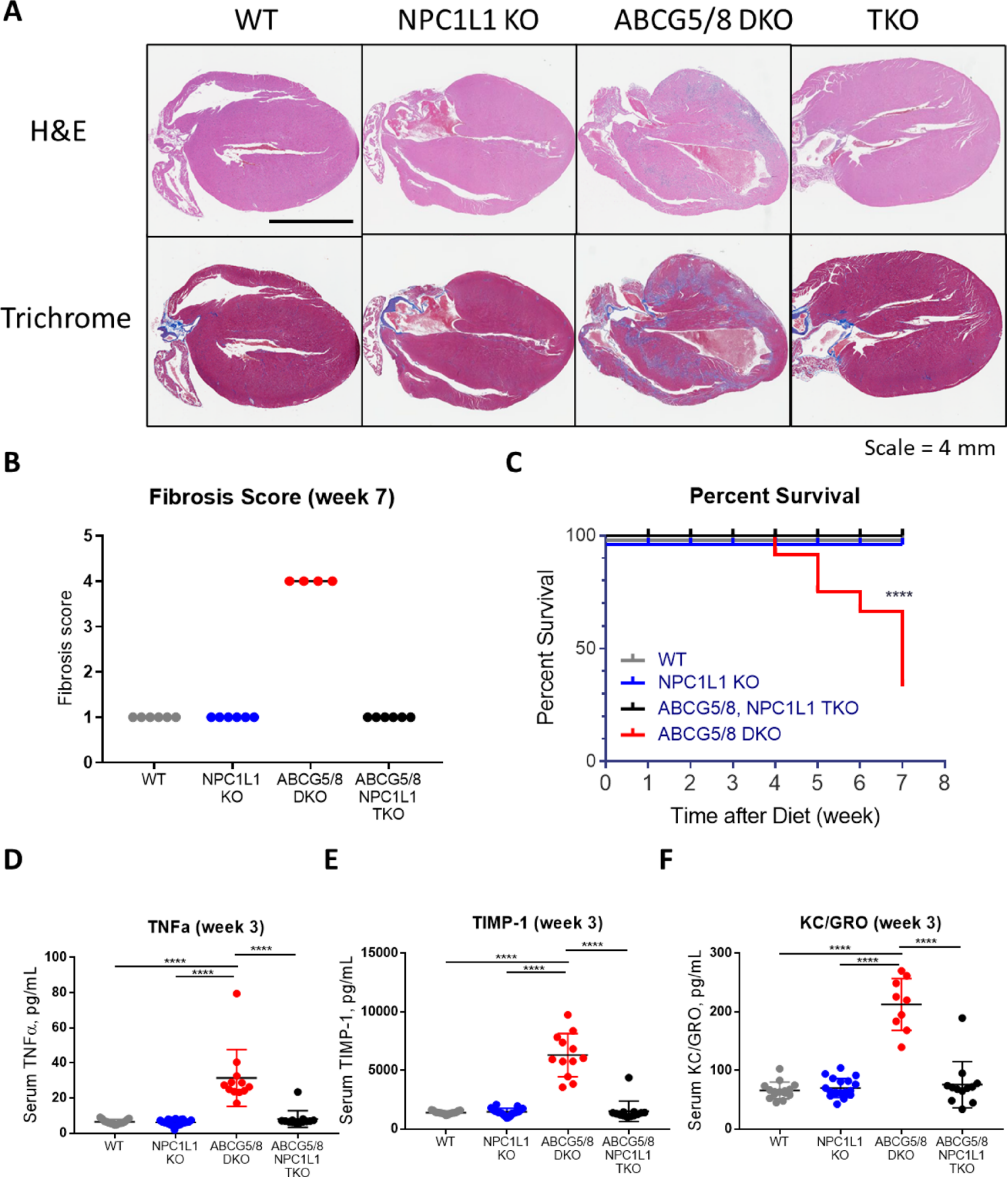
The NPC1L1, ABCG5/8 TKO exhibits reduced cardiac fibrosis, incidence of death and inflammation relative to ABCG5/8 DKO littermates. (**A**) Representative images from WT, NPC1L1 KO, ABCG5/8 DKO and NPC1L1, ABCG5/8 TKO mice at 7 weeks following the switch to the phytosterol enriched diet are shown. Scale bar equal 4 mm. (**B**) Cardiac fibrosis was scored as 0-within normal limits, 1-minimal, 2-mild, 3-moderate, and 4-severe. (**C**) ABCG5/8 DKO mice died beginning 4 weeks post-initiation of the phytosterol enriched diet. In contrast, the phytosterol enriched diet did not result in death in the TKO, NPC1L1 KO or WT littermate controls out to week 7 post-diet induction. Severe fibrosis in the ABCG5/8 DKO group at week 7 was associated with a significant increase in several inflammatory markers (serum TNF-α (**D**), TIMP-1 (**E**) and KC/GRO (**F**)) at week 3. DKO—ABCG5/8 double knockout, TKO—ABCG5/8, NPC1L1 triple knockout. Individual data points (circles) ± SEM are shown. WT: N = 15/ group; NPC1L1 KO: N = 21/group; TKO: N = 13/group; DKO: N = 12/group. Significance for panels D, E and F were measured by one-way ANOVA—Tukey post-test (*P < 0.05; **P < 0.01; ***P < 0.001; ****P < 0.0001). Significance for survival analysis, panel C, was measured by a log-rank Mantel-Cox test (****P < 0.0001). Histo-pathological assessment performed blinded.

Histopathological assessment of hearts after 7 weeks of phytosterol diet-challenge revealed that NPC1L1, ABCG5/8 TKOs exhibited significantly lower cardiac fibrosis relative to ABCG5/8 DKO littermates. Fibrotic scores from H&E and trichrome stained hearts were comparable for the NPC1L1, ABCG5/8 TKOs when compared to either NPC1L1 KOs or wild type littermates. This contrasts the ABCG5/8 DKO group, which exhibited a fibrotic score of 4 on a 0–4 point scale indicating that the hearts in this group contained diffusely distributed fibrosis in the left and right ventricles as well as the interventricular septum, which were separated and often compressed in individual cardiomyocytes. Fibrotic areas contained mature and immature collagen with active fibroblasts mixed with inflammatory cells (macrophages, lymphoid cells, and rarely neutrophils) indicating an actively ongoing fibrotic process. (Fig. 3A,B, Supplemental Fig. 2). Elevated circulating phytosterols and cardiac fibrosis was associated with an increased incidence of death for ABCG5/8 DKO mice, while the reduction in phytosterols and cardiac fibrosis corresponded to a reduction in mortality for NPC1L1, ABCG5/8 TKO littermates (Fig. 3C). ABCG5/8 DKO mice began to die after 4 weeks of phytosterol-enriched diet challenge with only 4 out of 12 mice (30%) remaining after 7 weeks. This was preceded by a decline in body weight for the ABCG5/8 DKO group (Supplemental Fig. 3). The degree of cardiac fibrosis and the increased incidence of death also correlated with circulating inflammatory cytokine levels at week 3 (Fig. 3D,F). TNFa, TIMP-1, KC/GRO were all significantly elevated for the ABCG5/8 DKO group, consistent with phytosterolemia eliciting an inflammatory response triggering fibroblast activation and fibrosis. Collectively, these data indicate that NPC1L1 inactivation compensates for the loss of ABCG5/8 by attenuating phytosterol accumulation thereby reducing inflammation, cardiac fibrosis and mortality.

### The NPC1L1 inhibitor, ezetimibe, only partially inhibits phytosterol transport

**Figure 4:**
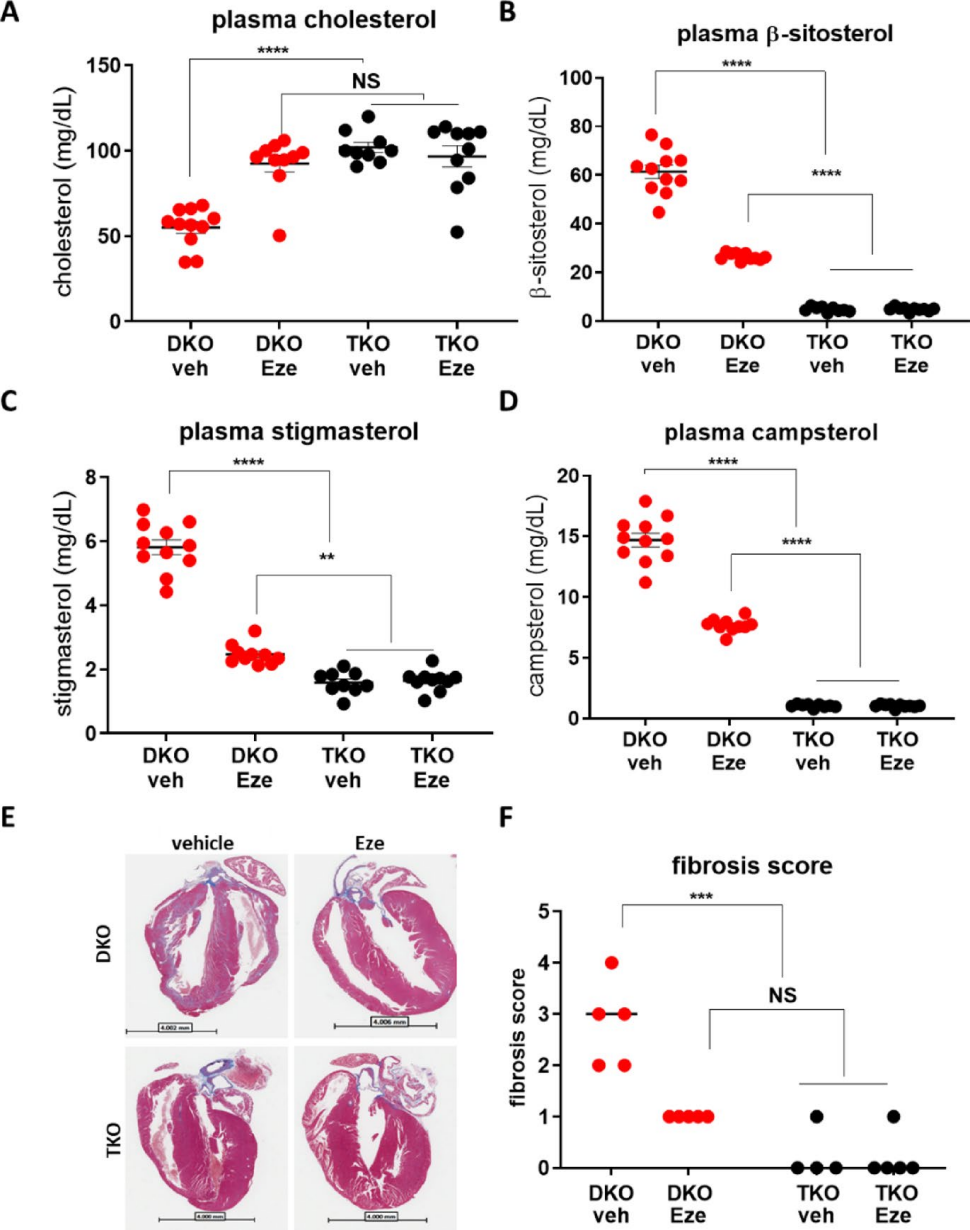
The NPC1L1 inhibitor, ezetimibe, partially prevents ABCG5/8 deficiency-derived phytosterolemia. Ezetimibe treatment led to increased plasma cholesterol (**A**) and decreased phytosterols (β-sitosterol (**B**), stigmasterol (**C**) and campsterol (**D**) in ABCG5/8 DKO mice. Plasma levels of phytosterols were lower in the NPC1L1, ABCG5/8 TKO relative to the ABCG5/8 DKO littermates treated with ezetimibe. Both ezetimibe and NPC1L1 KO decreased ABCG5/8 DKO induced fibrosis (**E**, **F**). DKO—ABCG5/8 double knockout, TKO—ABCG5/8, NPC1L1 triple knockout, veh—vehicle, Eze—ezetimibe. Individual data points (circles) are shown together with the sample mean (bar) ± SEM. Significance measured by two-way ANOVA—Sidak post-test (NS—not significant; *P < 0.05; **P < 0.01; ***P < 0.001; ****P < 0.0001). Histo-pathological assessment performed blinded.

To determine if the NPC1L1 inhibitor, ezetimibe, is sufficient to completely inhibit phytosterol transport, we compared plasma phytosterol levels in NPC1L1 KO mice to mice treated with a maximum efficacious dose of ezetimibe (10 mg/kg, QD) for 3 weeks. Fifteen week-old littermates were placed on the phytosterol enriched diet and began daily ezetimibe or vehicle treatment (p.o.) on day 0. Three weeks of daily ezetimibe administration resulted in a significant reduction in plasma phytosterols and a significant increase in plasma cholesterol as measured by a two-way ANOVA—Sidak’s post-test, comparing the two independent variables, genotype and treatment (Fig. 4A–D). Genetic inactivation of NPC1L1 resulted in a greater reduction in circulating phytosterols relative to ezetimibe treatment as evident by the significantly lower plasma phytosterol levels in NPC1L1, ABCG5/8 TKOs relative to ABCG5/8 DKO littermates treated with ezetimibe. Reducing plasma phytosterol levels either by genetic inactivation of NPC1L1 or through NPC1L1 inhibition with ezetimibe led to a significant reduction in cardiac fibrosis (Fig. 4E,F, Supplemental Fig. 4).

### Phytosterolemia led to inflammation and arrhythmia pathway induction

**Figure 5:**
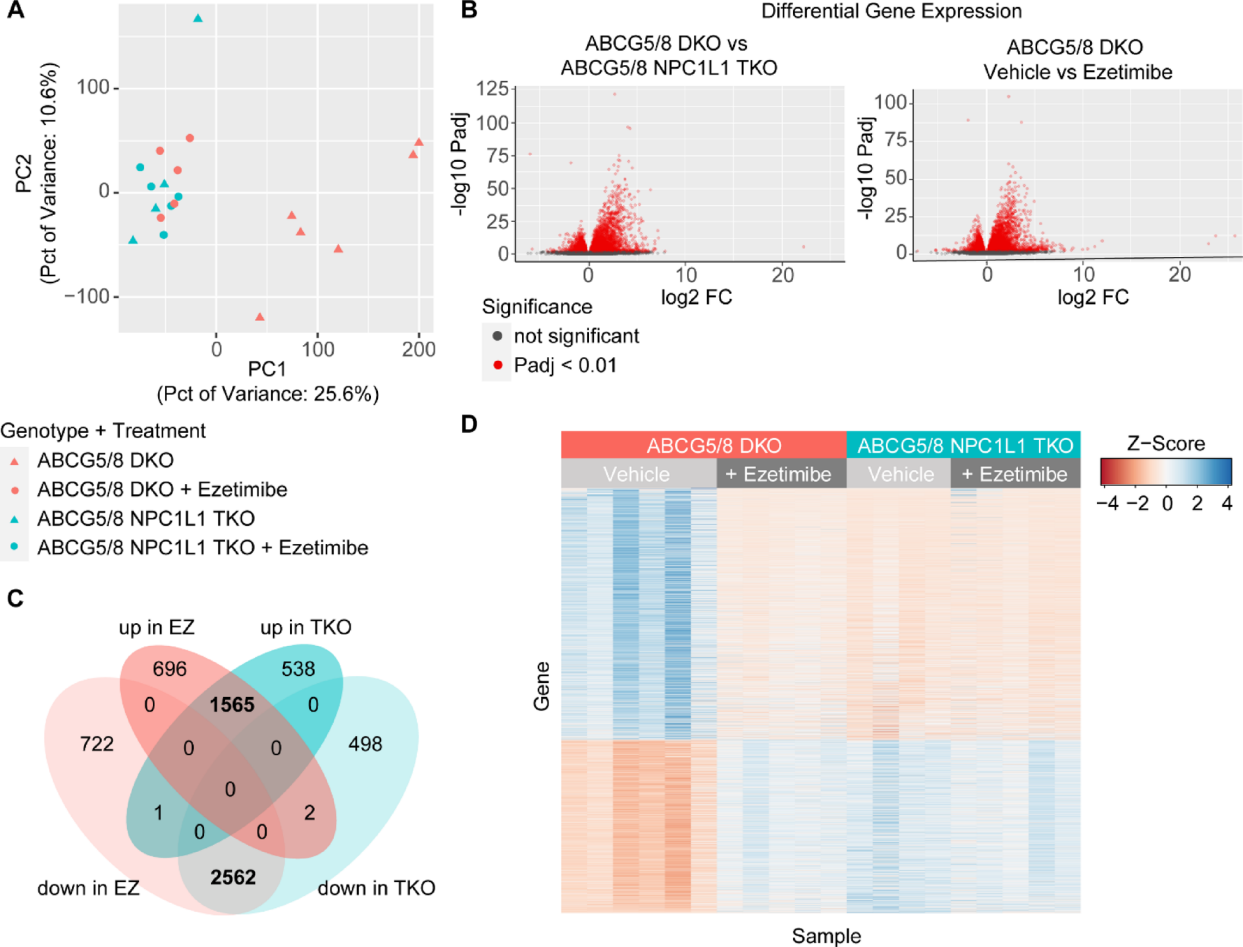
RNA-seq analysis of heart samples. (**A**) Plot of first two components of principal component analysis (PCA) of gene expression. ABCG5/8 double knockout (DKO) samples are filled with red and ABCG5/8 NPC1L1 triple knockout (TKO) samples are filled with cyan. Vehicle samples are triangles and ezetimibe treated samples are circles. (**B**) Volcano plots of *P* values from differential expression analysis versus log2 fold change in gene expression as calculated using DEseq2 comparing DKO untreated to TKO or DKO ezetimibe treated samples. A positive fold change value indicates higher expression in ABCG5/8 DKO untreated samples. (**C**) Venn diagram demonstrating overlap in differentially expressed (DE) genes from two comparisons. (**D**) Heatmap of gene expression (quantile normalized FPKM) Z-scores of DE genes identified comparing DKO and TKO conditions. DKO + vehicle: N = 6/group; DKO + ezetimibe: N = 5/group; TKO + vehicle: N = 5/group; TKO + ezetimibe: N = 5/group.

To explore phytosterolemia induced changes in gene expression, we next examined the gene expression profiles from 21 cardiac tissue samples that were collected from ABCG5/8 DKO and ABCG5/8, NPC1L1 TKO mice three weeks after the simultaneous placement on a phytosterol-enriched diet and initiation of either ezetimibe (10 mg/kg, QD) or vehicle control treatment. To explore the similarity in gene expression across conditions, we performed a principal component analysis (PCA) and observed a separation from ezetimibe and vehicle treated DKO samples in the first principle component (PC1), which explained 25.6% of the variance. The DKO ezetimibe treated cardiac samples clustered with TKO ezetimibe and vehicle treated cardiac tissue samples (Fig. 5A).

We next identified differentially expressed (DE) genes in DKO vehicle treated samples relative to DKO ezetimibe treated and TKO cardiac tissue samples. DEseq2 analysis using a cutoff of padj < 0.01 uncovered 5,548 DE genes in DKO ezetimibe treatment and 5,166 DE genes in the TKO relative to vehicle treated DKO (Fig. 5B, Supplemental Table 1). DE genes from the two analyses demonstrated high overlap with 1,565 shared genes significantly upregulated and 2562 genes downregulated in both DKO ezetimibe treated and TKO vehicle treated samples. Furthermore, we observed high consistency in the directionality of expression and few inverse effects in the two comparisons with only 3 DE genes with significant upregulation in DKO ezetimibe treated or TKO samples and downregulated in the other comparison (Fig. 5C). Overall, the comparison between differentially expressed genes from the DKO vs. TKO-vehicle, DKO vs. DKO-ezetimibe and DKO vs. TKO-ezetimibe samples exhibited a high degree of overlap to one another (Fig. 5D).

**Figure 6:**
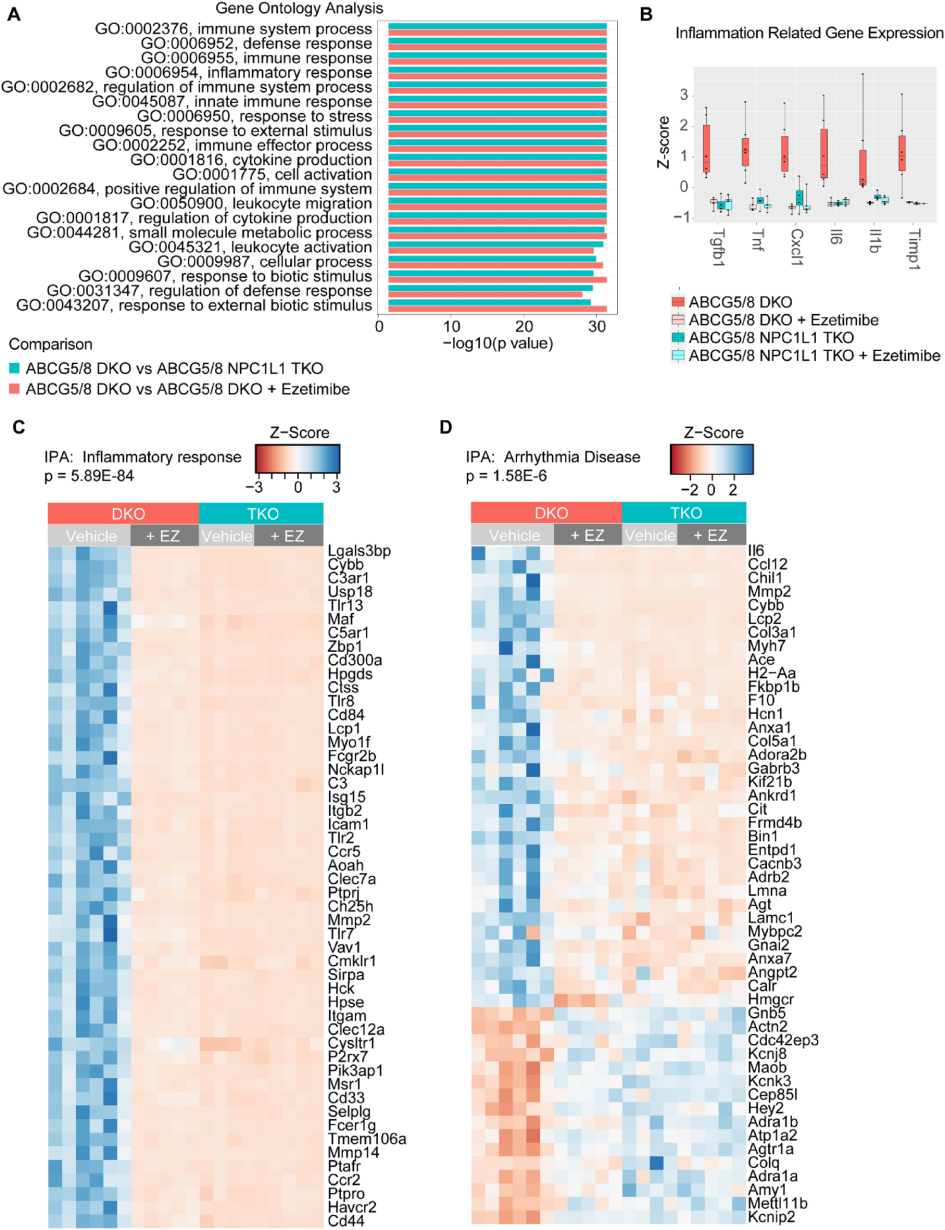
Functional analysis of RNA-seq data. (**A**) Gene Ontology (GO) enrichment analysis of differentially expressed (DE) genes comparing ABCG5/8 double knockout (DKO) samples to ABCG5/8 NPC1L1 triple knockout (TKO) (cyan) or DKO ezetimibe (red) treated samples. *P* values for the top twenty GO categories from the DKO/TKO comparison are plotted. (**B**) Box plots of gene expression (quantile normalized FPKM) Z scores of selected inflammation related genes. Scores of individual samples are overlaid on plots as points. (**C**) Heatmap of gene expression of 50 DE genes with the highest significance in the IPA Inflammatory Response and Arrythmia Disease (**D**) categories. DKO + vehicle: N = 6/group; DKO + ezetimibe: N = 5/group; TKO + vehicle: N = 5/group; TKO + ezetimibe: N = 5/group.

With DE genes identified, we next explored functional pathways affected by ezetimibe treatment or NPC1L1 gene inactivation using gene ontology (GO) enrichment analysis and Ingenuity Pathway Analysis (IPA). We observed a high degree of overlap in GO terms enriched in the comparisons. Notably, many of the highly significant GO terms are related to immune function and inflammation such as GO:0002376, immune system process [*P* < 1e−30 both comparisons] and GO:0006954, inflammatory response [*P* < 1e−30 both comparisons] (Fig. 6A, Supplemental Table 1). Taking a closer look at candidate inflammation-related genes, we observed upregulation of these genes in the vehicle treated DKO cardiac tissue samples relative to ezetimibe treated DKO, ezetimibe and vehicle treated TKO cardiac tissue samples (Fig. 6B). Similarly, IPA analysis identified significant enrichment of inflammatory response pathways [*P* = 5.89e−84 (DKO v TKO), 5.48e−83 (DKO vs treated)] and gene expression of DE genes in this category also showed upregulation in DKO vehicle treated samples relative to ezetimibe and vehicle treated TKO samples (Fig. 6C). Additionally, in the IPA analysis, the arrhythmia disease category demonstrated significant enrichment [*P* = 1.58e−6 (DKO v TKO), 3.69e−6 (DKO vs treated)] with similar gene expression patterns of DE genes in ezetimibe treated DKO and TKO samples (Fig. 6D).

### Phytosterols and cardiac arrhythmia

**Figure 7:**
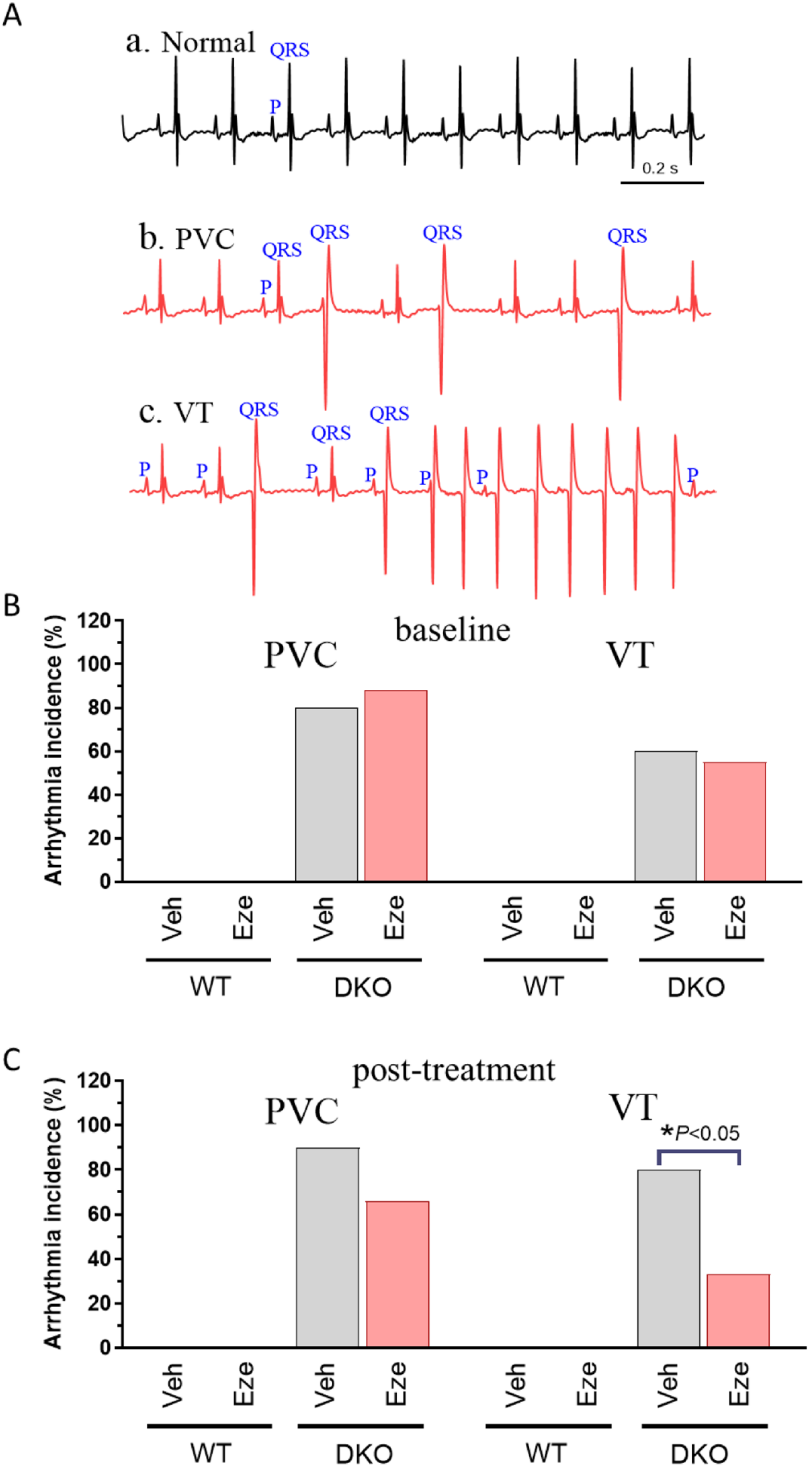
Ezetimibe reduces incidence of spontaneous ventricular arrhythmias in ABCG5/8 DKO mice. (**A**) Spontaneous ventricular arrhythmias in ABCG5/8 DKO mice. ***a***. normal sinus rhythm; ***b***, premature ventricular contraction (PVC); ***c***, ventricular tachycardia (VT). Incidence of ventricular arrhythmias at baseline (**B**) and after vehicle or ezetimibe treatment (**C**). Statistical significance for the incidence of arrhythmias between groups was determined using a Chi-Square test.

We hypothesized that increased ventricular arrythmias may be a cause of death for ABCG5/8 DKO mice with phytosterolemia. We evaluated the hypothesis by analyzing ECG recordings collected from anesthetized 22–26 week-old ABCG5/8 DKO mice and wild type littermates. Recordings were collected at baseline. Animals were then simultaneously placed on the phytosterol enriched diet and began either daily ezetimibe (10 mg/kg) or vehicle treatment (QD, p.o.). Recordings were collected again after 4 weeks. Wild type mice were free of ventricular arrhythmias both at baseline and at 4 weeks post-treatment. In contrast, we observed a high level of PVCs and VTs in DKO mice prior to placement on the phytosterol enriched diet. Over 80% of ABCG5/8 DKO mice have premature ventricular contractions (PVC), and over 55% of ABCG5/8 DKO mice have ventricular tachycardia (VT) at baseline (Fig. 7A,B). It is important to note that phytosterols are present in normal chow, and the animals are ingesting phytosterols prior to exposure to the phytosterol enriched diet, which acts to accelerate disease progression. Additionally, the mice used for the ECG studies were older when compared to the other studies we conducted (22–26 weeks vs 12–15 weeks), which may influence disease severity. At the week 4 time point ezetimibe treated mice exhibited a slight decrease in PVCs (90% vs 66%) but a significant reduction in VTs (80% vs 33%) when compared to DKO vehicle treated mice (Fig. 7C).

## Discussion

**Figure 8:**
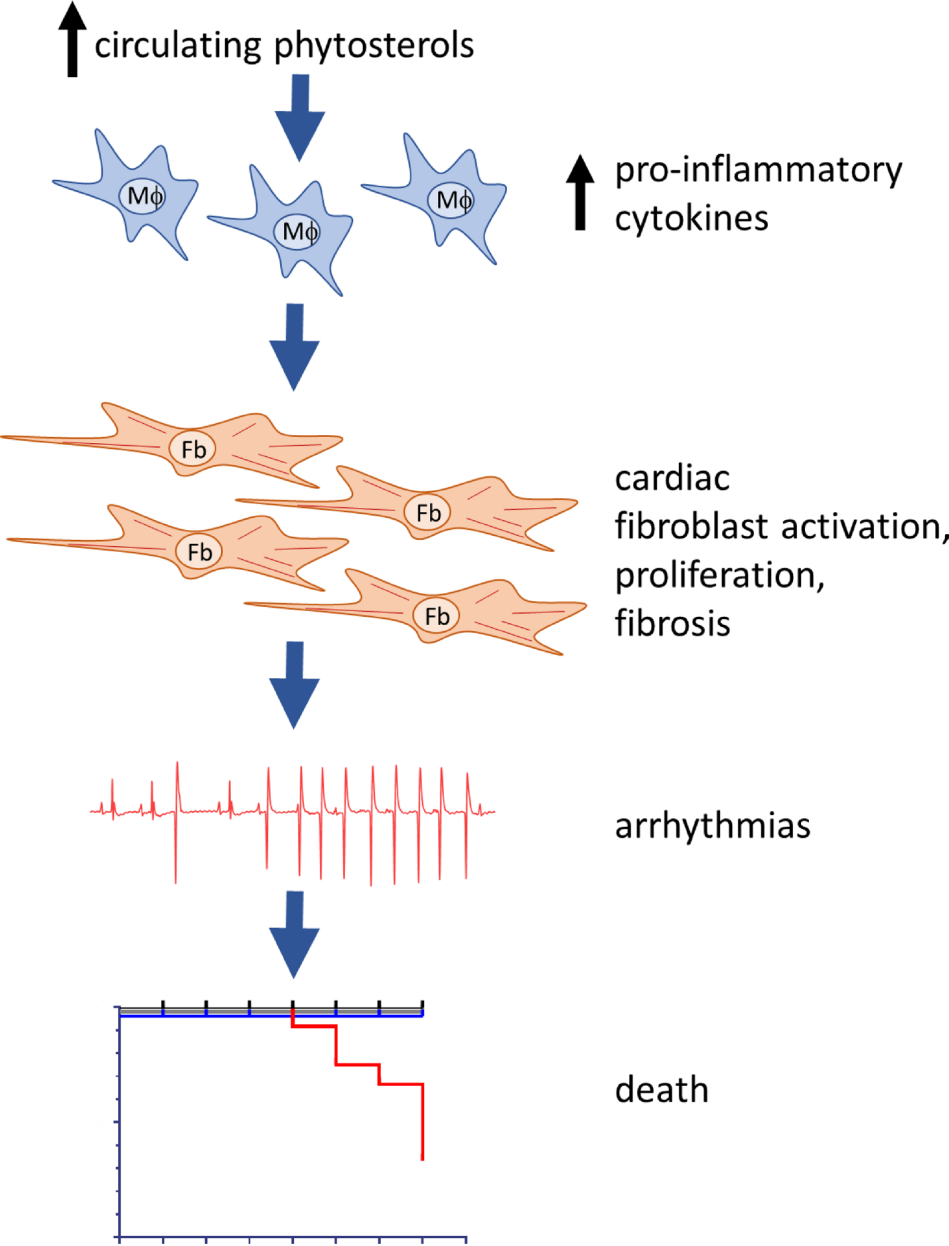
Our data support a model where elevated circulating phytosterols trigger inflammation, leading to fibroblast activation that cause structural rearrangements to the heart causing cardiac injury, arrhythmia and death.

We extended our previous study which revealed that phytosterolemia could lead to cardiac fibrosis resulting in premature death^23^. Here, we show that phytosterolemia led to an increase in the incidence of ventricular arrhythmias. Genetic inactivation of NPC1L1 or inhibition with ezetimibe reduced blood phytosterols, inflammation, cardiac fibrosis, ventricular arrhythmias and sudden cardiac death. Through the identification of several novel phytosterolemia associated comorbidities, we now have a better understanding of the underlying pathophysiology that links phytosterolemia to increased mortality in mice. We have shown that the accumulation of phytosterols results in chronic inflammation, cardiac fibrosis, cardiac injury, arrhythmias and death. Our data support a model where phytosterols accumulate in circulation triggering inflammation that results in fibroblast activation. This activation is associated with structural rearrangements to the heart causing cardiac injury and sudden cardiac death (Fig. 8).

The high incidence of PVCs and VTs is likely the result of fibrosis-induced changes in the molecular architecture of cardiac tissue where individual cells were frequently observed to be separated and compressed. The normal electrical connectivity of cardiac tissue is disrupted and the vulnerability to arrhythmias is increased^33–35^. These anatomical and functional changes have been shown to alter normal cardiac electrical conduction pathways and may explain why phytosterolemia patients are at increased risk of experiencing a myocardial infarction or sudden cardiac death as arrhythmia is often attributed to be the underlying cause^11,36–38^.

Phytosterolemia caused by deleterious mutations within the ABCG5/8 heterodimeric transporter is estimated to affect more than 1 in 200,000 individuals within the general population^36^. Patients with phytosterolemia exhibit elevated circulating phytosterols. Additionally, phytosterol deposition in tissues can reach more than one hundred times normal levels in these individuals. They are also at increased risk of developing cardiovascular disease.

Both phytosterolemia and hypercholesterolemia are commonly described as cardiovascular disorders that are generally assumed to lead to cardiovascular disease through similar pathological mechanisms. While cardiovascular disease is typically driven by hypercholesterolemia-induced vascular atherosclerosis, our work uncovered a distinct pathological mechanism in mice for phytosterolemia-induced cardiovascular related death that is driven by cardiac fibrosis and associated arrhythmias. While there are no known reports of either cardiac damage or ventricular arrhythmias contributing to cardiovascular outcomes for phytosterolemia patients, the His250Tyr coding variant’s association with aortic valve stenosis, heart failure and sudden cardiac death suggests a possible contribution to cardiac injury and mortality. These associations together with our observations in a mouse model of phytosterolemia suggest that an examination of cardiac tissue morphology using cardiac MRI or a related technique as well as screening for ventricular arrhythmias may be warranted.

Phytosterolemia patients are sometimes misdiagnosed with severe hypercholesterolemia as standard analytical methods used to quantify cholesterol are unable to distinguish cholesterol from structurally related phytosterols^23^. This would help to explain why some patients diagnosed with hypercholesterolemia respond poorly to statin therapy, as statins, which inhibit cholesterol synthesis, do not lower circulating phytosterols, as they are solely acquired by dietary absorption^11,39,40^.

Our data indicate that blocking phytosterol absorption through NPC1L1 inhibition can reduce inflammation, cardiac fibrosis, ventricular arrhythmia and death in mice. If our observations in mice translate to phytosterolemia patients, then it will underscore the importance of raising awareness of this disorder, considering the issues surrounding misdiagnosis, the fact that statins are the first line standard of care therapy for hypercholesterolemia and are known to provide limited clinical benefit for phytosterolemia patients relative to the NPC1L1 inhibitor, ezetimibe^36,41–44^.

Our work additionally reveals that a maximum efficacious dose of ezetimibe does not completely inhibit NPC1L1 mediated phytosterol transport in mice with phytosterolemia. The reduced but significantly elevated phytosterols in ABCG5/8 DKO-Eze cohort following a maximum efficacious dose of ezetimibe in comparison with TKO mice may lead to a more gradual development of cardiac fibrosis that was not detectable within the duration of our studies. Given that NPC1L1 genetic inactivation resulted in even lower phytosterol levels raises the possibility that a more potent inhibitor of NPC1L1 mediated phytosterol transport could lower phytosterol levels further and be a more efficacious therapy to treat phytosterolemia. It also raises the possibility that targeting ABCG5/8 with an agonist may represent a particularly valuable therapeutic strategy for phytosterolemia patients.

## Materials and methods

### In vivo

All studies reported in this manuscript follow the recommendations in the ARRIVE guidelines (https://arriveguidelines.org). All rodent studies were conducted at Amgen Inc and were approved by the Amgen Institutional Animal Care and Use Committee (IACUC) and complied with the Final Rules of the Animal Welfare Act regulations (Code of Federal Regulations, Title 9), the Public Health Service Policy on Humane Care and Use of Laboratory Animals in the Office of Laboratory Animal Welfare (2002), and the Guide for the Care and Use of Laboratory Animals from the National Research Council (1996). Mice were housed in ventilated cages on corncob bedding with 12:12 h light:dark cycle. C57B/6-NPC1L1 KO and C57B/6-ABCG5/ABCG8 DKO (B6;129S6-Del(17Abc5-Abc8)1Hobb/J) mice were licensed from Mount Sinai and UT Southwestern, respectively^32,45^. Both lines were backcrossed to C57Bl/6 mice obtained from Charles River to achieve 99–100% homozygosity. The NPC1L1*,* ABCG5/8 TKOs were generated by crossing mice that were heterozygous KOs for both ABCG5/8 and NPC1L1 to each other (Het/Het x Het/Het). Mice were given ad libitum access to food and water. For ezetimibe administration, vehicle or 10 mg/kg ezetimibe (Fisher, 50-753-2773, Cat#163222-33-1) were administered daily via oral gavage for 3 weeks. Mice were euthanized and tissues were excised and fixed in 10% buffered formalin overnight and then transferred to 70% ethanol.

### Echocardiography

Non-invasive echocardiograms were obtained on conscious mice using a Vevo 2100 imaging system. Hair was removed using commercially available depilatory cream the day before, mice were placed on a stage and sonography gel was applied on the thorax. Two-dimensional targeted ‘M-mode’ imaging was obtained from the short-axis view at the level of the papillary muscle. Animals were subsequently wiped clean of sonography gel and returned to their home cage.

### Electrocardiogram (ECG)

The mice were induced with 3% isoflurane and maintained with 1–1.5% isoflurane delivered with COMPAC5 vaporizer. Loss of the right limb reflex by applying a strong toe pinch was used to judge the depth of the anesthesia plane before starting the procedure. Mice were then placed in a dorsal recumbence position on a heated pad. The limbs were fixed with tape. After ECG recording, mice were allowed to wake up prior to being returned to their cage.

A six lead (I, II, III, aVF, aVL, aVR) electrocardiogram (ECG) was recorded using a digital acquisition system (Biopac, MP160) from subdermal ECG needle electrodes (29 gauge, 12 mm long; AD Instruments) inserted subcutaneously into the left and right forelimbs and into the left hindlimb. The surface ECG tracings were filtered using a high pass setting of 1 Hz and a low pass setting of 150 Hz. The signal was acquired for 5 min using AcqKnowledge 5 software.

ECG was manually analyzed offline. Arrhythmic events, specifically, premature ventricular contraction (PVC) and ventricular tachycardia (VT) were carefully examined to determine the arrhythmia incidence in mice. PVC was counted as positive for a mouse when the average PVC occurrence is ≥ 1 PVC/per minute. The occurrence of 3 consecutive ventricular beats was counted as VT positive. A Chi-Square Test was used to determine the statistical significance of incidence between groups.

#### LC–MS/MS

Plasma samples were analyzed using liquid chromatography with tandem mass spectrometry (LC–MS/MS, performed by Metabolon Inc., Durham, NC) for the contents of the following sterols (after hydrolysis of sterol esters): cholesterol, β-sitosterol, campesterol, and stigmasterol. Sample analyses were carried out in a 96-well plate format containing two calibration curves and six QC samples (per plate) to monitor method performance. The mass spectrometer was operated in selected ion monitoring (SIM) mode. The masses of the extracted ions were 458.4 (cholesterol), 382.4 (campesterol), 396.4 (β-sitosterol), and 523.2 (stigmasterol). *Histology*—Following paraffin embedding, tissue sections were stained with hematoxylin and eosin (H&E) (Biocare IPCS5006G20), and trichrome using standard protocols. Images were obtained using Aperio Digital Pathology Slide Scanner (Leica Biosystems) and Aperio ImageScope v12 software. The evaluation of slides was done by board-certified veterinary pathologist based on following scoring criteria: (0) no fibrosis, (1) minimal fibrosis (barely noticeable interstitial or focal replacement fibrosis), (2) mild fibrosis (easily detectable multifocal interstitial or replacement fibrosis), (3) moderate fibrosis (consistently observed in all chambers and interconnected lattice of fibrosis), (4) severe fibrosis (same as 3 with large fibrotic areas of replaced cardiac tissue).

### Circulating cytokines

Serum TNF-alpha, KC/GRO and TIMP-1 concentrations were measured at week 3 using the MSD V-PLEX Cytokine Panel 1 Mouse Kit (K15245D) and the R&D Systems Mouse TIMP1 Quantikine ELISA Kit (MTM100), respectively. These assays were performed as described within the product protocols.

### Bulk RNA Seq and associated analyses

150 ng of total RNA with ERCC spike-ins was used as input for the TruSeq Stranded mRNA Library Prep (Illumina). Libraries were sequenced on an Illumina HiSeq4000 to a minimum depth of 30 million 150 × 150 paired-end reads. Alignment and quantification were performed using Oshell v10.0.1.111^46,47^. Reads were aligned to the OmicsoftGencode.V19 mouse reference based on GRCm38. Quantile normalization of FPKM values was performed using the 70th percentile of gene expression according to the following formula: FPKQ = 10 FPKM/(FPKM 70th Percentile)^48^.

Principal component analysis (PCA) was performed using the prcomp function in R on centered and scaled log transformed FPKQ data of genes detected in at least one sample. This analysis identified a single outlier (TKO no treatment sample) that was removed from downstream analyses. DEseq2 v1.26.0 was used to identify differentially expressed (DE) genes, DKO untreated vs treated or DKO vs TKO, using count data and the Wald test to determine *P* values^49^. Gene Ontology enrichment analysis was performed on DE gene analysis *P* values using the topGO package v2.31.1^50^. Further functional analysis of DE genes was performed using IPA (QIAGEN Inc., https://www.qiagenbioinformatics.com/products/ingenuity-pathway-analysis;^51^).

### Quantification and statistical analysis

All data are reported as the mean ± S.E.M. The differences between the mean values were tested for statistical significance by one-way ANOVA as indicated in the figure legends.

## Supplementary Information

Supplementary Information 1.

Supplementary Information 2.

## Data Availability

All data generated and analyzed during this study are included in this article and supplementary information files. Sequencing data has been deposited in GEO under the accession GSE173123. Additional data and information about this study are available from the corresponding author on request. Further information and requests for resources and reagents should be directed to the lead contact, Dr. Brandon Ason (bason@amgen.com).
