# Supplementary material for: Phytosterol accumulation results in ventricular arrhythmia, impaired cardiac function and death in mice
Source: Sci Rep. 2021 Aug 31;11:17449. doi: 10.1038/s41598-021-96936-x (PMC8408133; doi:10.1038/s41598-021-96936-x)
Supplement: Supplementary file 1 — Supplementary Information 1. [file 41598_2021_96936_MOESM1_ESM.docx]

**Supplemental Fig. 1. No significant difference in heart to brain weight ratios observed for the ABCG5/8 DKO relative to their littermates when fed a phytosterol enriched diet for 7 weeks.** DKO - ABCG5/8 double knockout, TKO – ABCG5/8, NPC1L1 triple knockout. Individual data points (circles) are shown together with the sample mean (bar) ± SEM. Significance measured by one-way ANOVA - Tukey post-test.


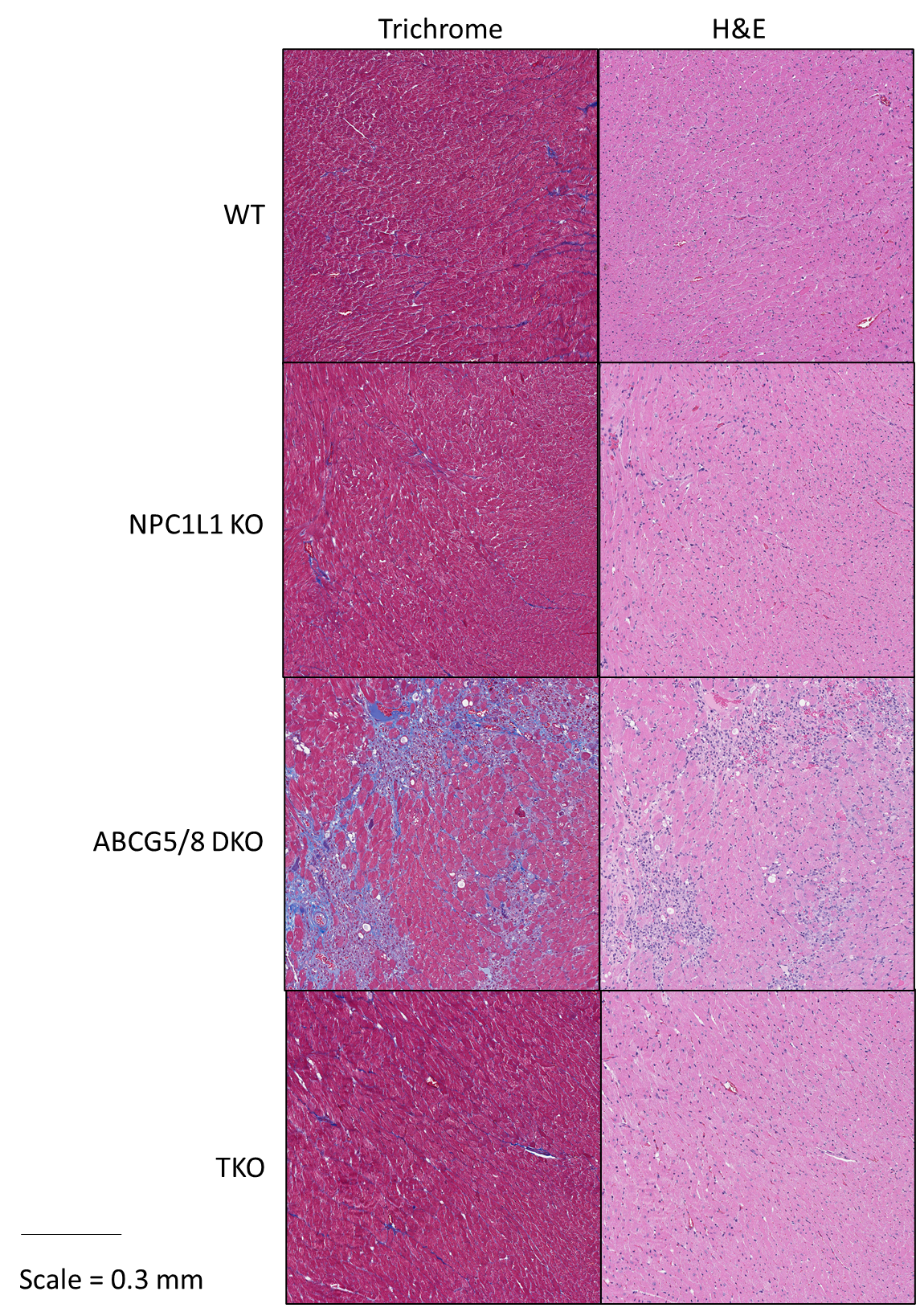


**Supplemental Fig. 2. Representative images of cardiac tissue ffrom WT, NPC1L1 KO, ABCG5/8 DKO and NPC1L1, ABCG5/8 TKO at 7 weeks following the switch to the phytosterol enriched diet are shown.** Scale bar equal 0.3 mm.

## Supplemental Fig. 3. The ABCG5/8, NPC1L1 TKO does not exhibit lower body weight observed in the ABCG5/8 DKO littermates. The ABCG5/8 DKO, C57Bl/6 WT, NPC1L1 KO, and ABCG5/8, NPC1L1 TKO littermates were 20 weeks old when they were placed on the phytosterol enriched diet (week 0). The DKO littermates weigh less prior to being placed on the phytosterol enriched diet. The difference in body weight for the DKO mice relative to their littermates became more pronounced following 7 weeks of phytosterol enriched diet feeding. DKO - ABCG5/8 double knockout, TKO – ABCG5/8, NPC1L1 triple knockout. WT: N = 15/ group; NPC1L1 KO: N = 21 / group; TKO: N = 13 / group; DKO: N = 12 /group when mice were switched to the phytosterol enriched diet.


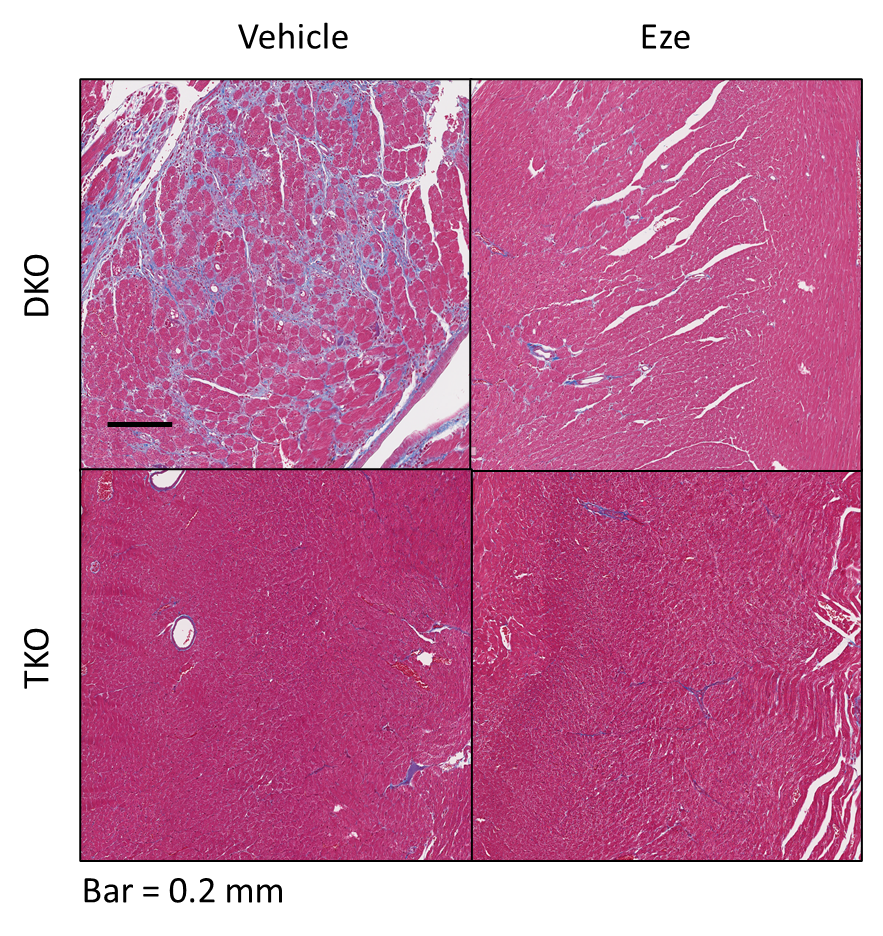


**Supplemental Fig. 4. Representative images of cardiac tissue from ABCG5/8 DKO and NPC1L1, ABCG5/8 TKO mice following 3 weeks of vehicle or ezetimibe (10 mg/kg, QD) treatment.** Scale bar equal 0.2 mm.
